# Supra-molecular assembly of a lumican-derived peptide amphiphile enhances its collagen-stimulating activity†
†Electronic supplementary information (ESI) available: DETAILS. See DOI: 10.1039/c5bm00428d



**DOI:** 10.1039/c5bm00428d

**Published:** 2015-12-02

**Authors:** Merlin N. M. Walter, Ashkan Dehsorkhi, Ian W. Hamley, Che J. Connon

**Affiliations:** a Institute of Genetic Medicine , International Centre for Life , Newcastle University , Newcastle upon Tyne , NE1 3BZ , UK . Email: che.connon@newcastle.ac.uk; b Department of Chemistry , University of Reading , Whiteknights , Reading , RG6 6AD , UK

## Abstract

C_16_-YEALRVANEVTLN, a peptide amphiphile (PA) incorporating a biologically active amino acid sequence found in lumican, has been examined for its influence upon collagen synthesis by human corneal fibroblasts *in vitro*, and the roles of supra-molecular assembly and activin receptor-like kinase ALK receptor signaling in this effect were assessed. Cell viability was monitored using the Alamar blue assay, and collagen synthesis was assessed using Sirius red. The role of ALK signaling was studied by receptor inhibition. Cultured human corneal fibroblasts synthesized significantly greater amounts of collagen in the presence of the PA over both 7-day and 21-day periods. The aggregation of the PA to form nanotapes resulted in a notable enhancement in this activity, with an approximately two-fold increase in collagen production per cell. This increase was reduced by the addition of an ALK inhibitor. The data presented reveal a stimulatory effect upon collagen synthesis by the primary cells of the corneal stroma, and demonstrate a direct influence of supra-molecular assembly of the PA upon the cellular response observed. The effects of PA upon fibroblasts were dependent upon ALK receptor function. These findings elucidate the role of self-assembled nanostructures in the biological activity of peptide amphiphiles, and support the potential use of a self-assembling lumican derived PA as a novel biomaterial, intended to promote collagen deposition for wound repair and tissue engineering purposes.

## Abstract 2

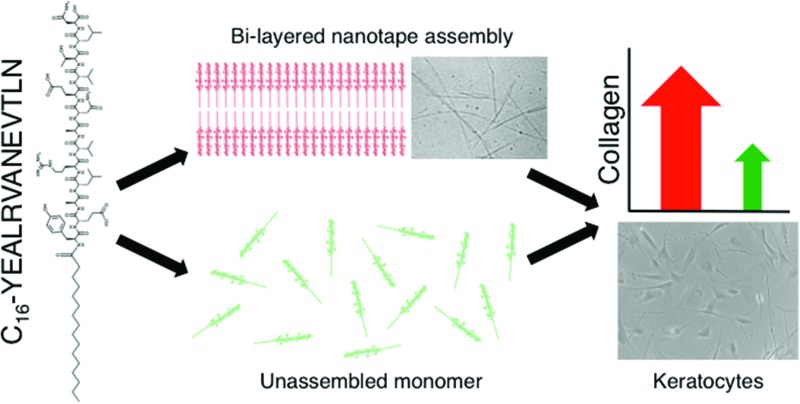
Lumican derived peptide amphiphiles can stimulate cells to produce greater amounts of collagen when used in an aggregated form. Moreover this effect is maintained following dilution suggesting that the aggregated forms are kinetically trapped when formed at high concentration.

## Introduction

Damage to the cornea, whether occurring through mechanical or chemical insult or as a result of hereditary or infectious disease, can result in reduced transparency and potential blindness. Corneal transplantation is the most common method of treatment once injury or disease has affected the clarity of the cornea, and whilst this is the most successful solid organ transplant performed, it is limited by its dependence upon the availability of suitable donor tissue.^1^ As a result of this, a large body of research has been focused on the engineering of corneal tissue suitable for therapeutic transplantation. Tissue engineering methods often seek to combine cells with a suitable scaffold to replicate native tissue. In addition to the synthetic scaffold materials and de-cellularised donor tissues commonly investigated for this purpose, recently there has been increased interest in the use of cells cultured *in vitro* to produce a native-like extracellular matrix (ECM) for subsequent seeding with tissue-specific cell types to potentially produce ‘organotypic’ engineered tissues. We, and others, have recently developed methods of culturing corneal stromal fibroblasts on synthetic substrates that inform or otherwise facilitate the production of collagenous lamellae intended to replicate corneal stroma.^2,3^ These techniques are dependent upon the *in vitro* synthesis of collagen by cells and could therefore benefit from factors that enhance or mediate this process. Additionally the healing of corneal wounds and conditions associated with pathological matrix degradation, such as keratoconus or chronic stromal ulceration, might benefit from the topical application of a bioactive molecule capable of stimulating collagen production by the endogenous cell population.

**Fig. 1:**
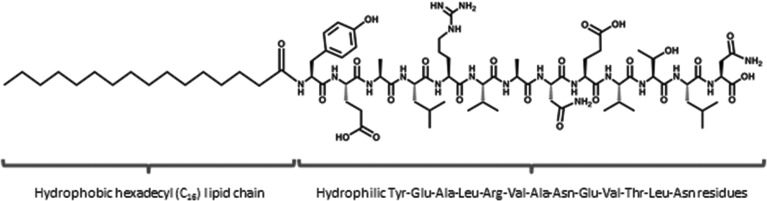
C_16_-YEALRVANEVTLN PA is comprised of a 13 amino acid sequence (Tyr-Glu-Ala-Leu-Arg-Val-Ala-Asn-Glu-Val-Thr-Leu-Asn) and a hexadecyl (C_16_) lipid chain.

Peptide amphiphiles are ‘a class of molecules that combine the structural features of amphiphilic surfactants with the functions of bioactive peptides and are known to assemble into a variety of nanostructures’,^4^ comprised of a hydrophobic ‘tail’ region and a hydrophilic peptide ‘head’. These molecules have demonstrated potential for a range of biomedical applications, either *via* their amphiphilicity and resultant compatibility with the lipid bilayer of cell membranes, capacity to present functional peptides in high densities upon the surface of supra-molecular assemblies, or suitability to form bioactive or cell-responsive ‘smart’ biomaterials,^3,5,6^ or a combination of these factors. Previously, a PA (C_16_-KTTKS) frequently used in commercially available skincare cosmetics, under the trade name Matrixyl™, was shown to stimulate the production of collagen by fibroblasts *in vitro*.^7^ Matrixyl is a lipidated peptide based upon a KTTKS pentapeptide head sequence, derived from a human type-1 collagen propeptide attached to a palmitic acid (C_16_) hexadecyl lipid chain.^8,9^ In this study we have investigated the effects of a PA based upon a 13-residue sequence, YEALRVANEVTLN, derived from the C-terminus of the small leucine rich proteoglycan lumican, also attached to a C_16_ hydrophobic tail (Fig. 1). In particular we focused on the enhanced bioactivity of the PA in supra-molecular form and its specific mode of action. Whilst there have been numerous publications studying the bioactivity of various PA's none, to the authors knowledge, have directly compared this activity of a PA in aggregated and unaggregated forms at the same concentration.

Lumican, along with keratocan and mimecan, is a major keratan sulphate proteoglycan found in the ECM of many collagenous tissues including skin, tendons, cartilage, and the corneal stroma.^10–12^ More specifically, in the cornea, lumican has been shown to be essential for the maintenance of corneal transparency^10,13^ by influencing epithelial and stromal cell behaviors though matrikine function *i.e.* stimulating corneal epithelial wound healing^14^ and regulating collagen fibrillogenesis.^15,16^ A recent study by Yamanaka and co-workers demonstrated the binding of lumican to the TGF-β superfamily type I activin receptor-like kinase, ALK5, by its C-terminal domain; an approximately 50 amino acid sequence. Within this sequence, a 13-residue peptide (YEALRVANEVTLN) capable of stimulating epithelial migration^17^ was identified. This work, as well as the collagen stimulating effects of another ECM-derived peptide sequence based PA (Matrixyl™), and the well-documented role of TGF-β signaling pathways in ECM synthesis and fibrotic response,^18^ has informed the current investigation. The peptide amphiphile C_16_-YEALRVANEVTLN, incorporating a biologically active amino acid sequence found in lumican, has been examined for its effects upon human corneal fibroblasts *in vitro*, and the contribution both of supra-molecular assembly and ALK signaling in these effects have been investigated.

## Materials and methods

### Materials

The C_16_-YEALRVANEVTLN PA was purchased from CS Bio (California, USA). Purity was 95.68%. The molar mass was 1730.89 Da (expected 1730.05 Da).

### Formation of monomeric and aggregated PA solutions

The PA was dissolved by ultra-sonication at 55 **°**C in ultra-pure water to concentrations either below (0.025 wt%) or above (0.1 wt%) the published critical aggregation concentration (CAC) 0.03 wt%, the point at which the PA self-assembles into a highly ordered twisted nanotape structure.^19^ At this point the PA exists as either a solution of monomeric molecules (in those samples made at 0.025 wt%) or as a solution of aggregated PA nanotapes (in those samples made at 0.1 wt%). These stock solutions (in monomeric or aggregated form; below or above the CAC) were each subsequently diluted in ultra-pure H_2_O to make 0.00125 wt% and 0.0025 wt% experimental solutions of PA, resulting in working solutions of equally concentrated but differently arranged PA molecules at each concentration, in order to compare the effects of supra-molecular assembly upon the cellular responses *in vitro*. Thus the supramolecular self-assembly of the PA at concentrations above its CAC is crucially maintained upon its subsequent dilution as aggregates formed above the CAC are kinetically trapped.

### Cell culture

Human corneal fibroblasts were isolated from corneal rings from post-mortem human donor eyes following depletion of epithelial cells. Corneal tissue was finely minced using a scalpel and subsequently digested in 2 mg ml^–1^ collagenase type-I (Invitrogen, USA) in Dulbecco's Modified Eagles Medium (DMEM) supplemented 5% fetal bovine serum (FBS) for 5 hours under gentle rotation (15 rpm) at 37 **°**C/5% CO_2_. Isolated cells were plated onto standard culture plates (Corning, USA) and maintained in DMEM/F12 media (Invitrogen) supplemented with 5% FBS, 1 mM ascorbic acid, and 1% penicillin/ streptomycin. The cells were routinely passaged at 70%–80% confluence and used experimentally between passage numbers 2–4. Culture medium was replaced with serum-free media (SFM) supplemented with 1% ITS (insulin, transferrin and selenium) (Invitrogen) three days prior to subsequent experiments in order to induce cells to adopt a keratocyte-like phenotype.

### Circular dichroism spectroscopy

Circular dichroism was performed according to previously published methods^20^ using a Chirascan spectropolarimeter (Applied Photophysics, UK). Spectra are presented with absorbance *A* < 2 at any measured point with a 0.5 nm step, 1 nm bandwidth, and 1 second collection time per step at 20 °C. Post-acquisition smoothing was used to remove random noise.

### Cell viability

The tolerance of human corneal fibroblasts to C_16_-YEALRVANEVTLN was determined using the Alamar blue assay, which correlates cellular metabolism to the reduction of a resazurin sodium salt (Sigma-Aldrich, Dorset, UK) by mitochondrial dehydrogenase activity. Fibroblastic cells, 1 × 10^4^ per well, were seeded (serum free) into 24 well plates (Corning) and allowed to adhere overnight before exposure to 0.00125 wt% and 0.0025 wt% solutions of PA (either in monomeric or aggregated form prior to dilution) in SFM. Cultures were maintained for seven days or three weeks. PA-supplemented and un-supplemented (control) growth media were replaced every two days over the culture periods. At the end of these culture periods media were replaced with resazurin reagent (1 : 10 in SFM) and incubated for 3 hours (37 **°**C/5% CO_2_) at which point 100 μl samples were taken in duplicate from each well and assayed for fluorescence emission at 590 nm under excitation at 544 nm using a Fluoroskan Ascent™ (Thermo scientific, Paisley, UK). The cell number was calculated by interpolation using a standard curve for the fluorescence values derived from known numbers of cells, determined in parallel with each separate assay. The PA solutions had no effect, in either monomeric or diluted aggregate form, upon the reduction of resazurin in the absence of cells.

### Collagen synthesis

The amounts of collagen produced by fibroblasts in response to the aforementioned concentrations of C_16_-YEALRVANEVTLN in momomeric or diluted aggregated form were assessed by the Sirius red assay. Following the evaluation of viable cell number by Alamar blue, seven day and three week fibroblast-cultures were fixed in ice-cold ethanol for 10 minutes at –80 **°**C and stained with 1 mg ml^–1^ Sirius-red/picric acid solution overnight. These wells were then repeatedly rinsed in phosphate buffered saline (PBS) and the bound dye was recovered after 15 minutes of agitation in 1 M NaOH. Total collagen content was calculated by comparing the absorbance of the resulting samples at 490 nm, read using a Multiskan Ascent™ spectrometer (Thermo scientific), to that of known standard concentrations of collagen (First Link, Wolverhampton, UK).

### ALK receptor inhibition

Sirius red assays were also performed upon cells treated with PA in the presence of an ALK inhibitor, SB 431542 (Sigma-Aldrich) at 0.5 μM in dimethyl sulfoxide (DMSO), compared to media supplemented with DMSO alone (vehicle only).

### Image capture and analysis

Digital images were captured using an Eclipse TS100 inverted microscope (Nikon, Surrey, UK) and a ProgRes speed XT core-5 digital camera (Jenoptik, Jena Germany).

### Statistical analysis

Data is presented as means of *n* = 3 independent experiments performed using cells from different donor corneas (±SEM) where each individual experimental value is the mean of *n* = 4 internal replicates. Data were tested for significance using a non-parametric ANOVA appropriate for non-normally distributed data^21^ and *post-hoc* analysis was performed using Tukey's HSD test.^22^ Those differences that fell within a 95% confidence interval were considered to be significant, indicated by asterisks within figures (*p < 0.05).

## Results

**Fig. 2:**
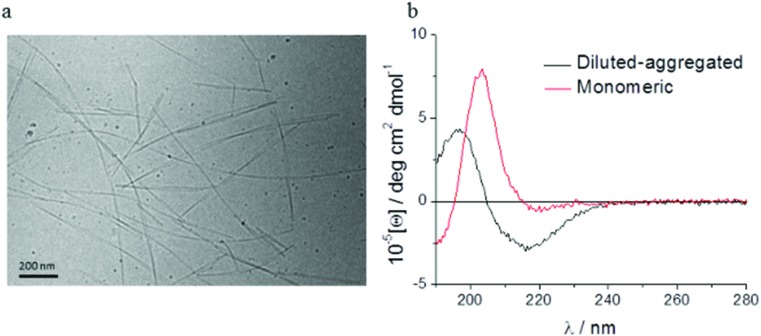
Cryo-TEM shows that aqueous solutions of C_16_-YEALRVANEVTLN PA form bi-layered nanotapes structures at 0.1 wt% concentrations (a). These secondary structures are maintained upon subsequent dilution to a biocompatible concentration of 0.0025 wt% (b) demonstrated by the minimum at 216 nm and the maximum at 195 nm typical of beta-sheet formations (black). These features are absent in spectra obtained from PA solutions initially prepared at 0.0025 wt% (red).

**Fig. 3:**
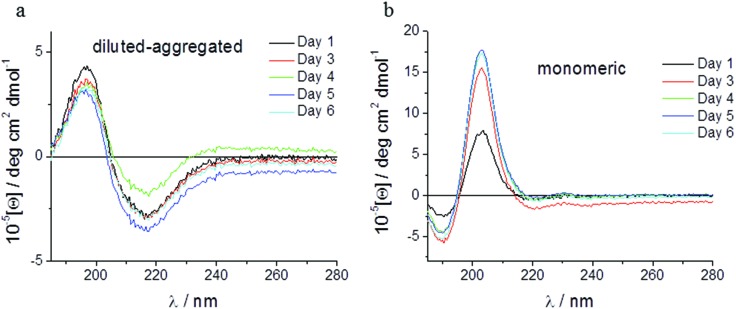
The secondary structure of C_16_-YEALRVANEVTLN PA is observable in solution for up to 6 days following dilution to 0.0025 wt%, as shown by circular dichroism spectroscopy, with no signs of degradation correlating to time in culture (a). At initial concentrations of 0.0025 wt%, aqueous solutions of C_16_-YEALRVANEVTLN PA do not display signs of secondary structure (b).

Spectroscopic methods were used to examine the secondary structure of the PA following its aqueous dilution from a sufficiently concentrated form (0.1 wt%) that results in the spontaneous self-assembly of bi-layered nanotape structures (Fig. 2a). Fig. 2b shows circular dichroism (CD) spectra for a 0.0025 wt% aqueous solution of PA either (i) initially made to 0.1 wt% (*i.e.* above its published CAC) and subsequently diluted to 0.0025 wt% henceforth referred to as diluted-aggregated (black) or (ii) made directly to 0.0025 wt% henceforth referred to as monomeric (red). The CD spectrum for (i) reveals a minimum at 216 nm and a maximum at 195 nm, characteristic of β-sheet secondary structure, that is not present in the spectra for (ii). This indicates the retention of secondary structures upon subsequent dilution of aggregated PA, and the absence of such in directly prepared (monomeric) solutions at 0.0025 wt%. Fig. 3a shows the CD spectrum for a 0.0025 wt% aqueous solution of PA diluted from an initial concentration of 0.1 wt% (aggregated form) and maintained over 6 days at room temperature. These spectra demonstrate that the secondary structure (initially formed by dissolving the PA above its CAC) persists for this period with no apparent degradation over time despite its subsequent dilution. Solutions of PA directly prepared to 0.0025 wt% (below the CAC) continue to display an absence of secondary structure throughout this period (Fig. 3b).

**Fig. 4:**
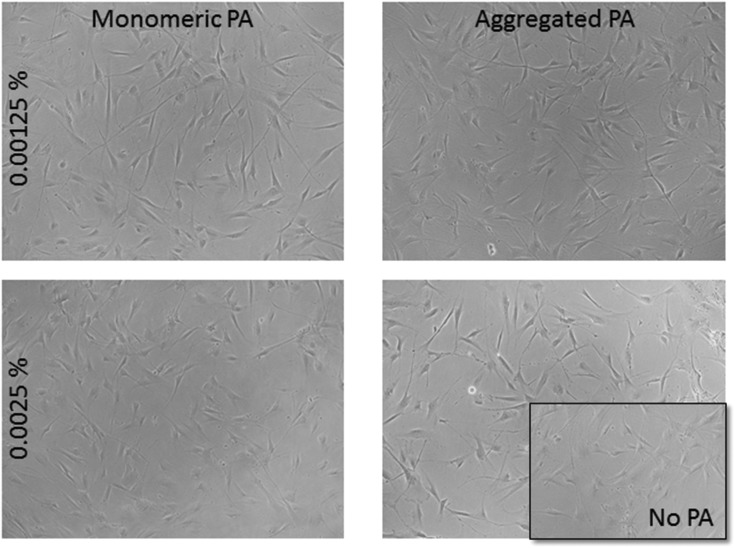
Fibroblasts tolerate PA concentrations of 0.00125 wt% and 0.0025 wt% and maintain normal morphology over 7 days in PA supplemented cultures.

**Fig. 5:**
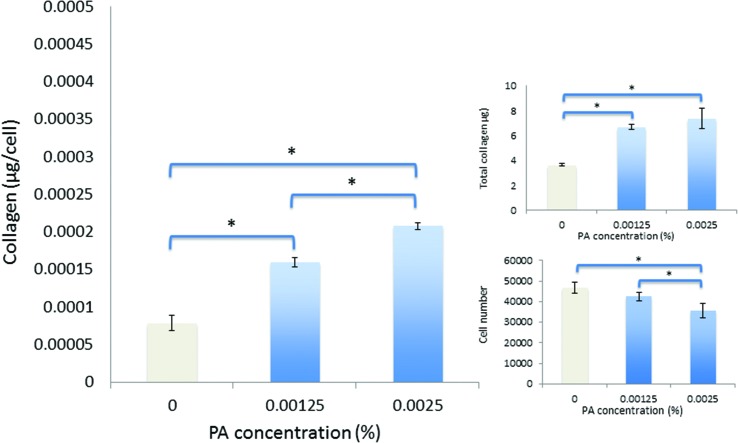
Viability and collagen synthesis by human corneal fibroblasts after culture for seven days in monomeric-PA supplemented SFM.

**Fig. 6:**
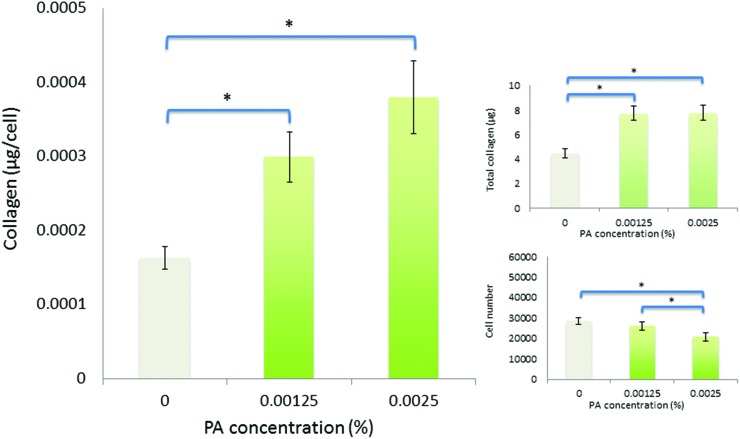
Viability and collagen synthesis by human corneal fibroblasts after culture for seven days in diluted-aggregated PA supplemented SFM.

Human corneal fibroblasts cultured in 0.0025 wt% and 0.00125 wt% PA solutions showed the normal fibroblastic morphology expected for cells in 2D culture (Fig. 4) and displayed good, though reduced, viability after seven days exposure to both monomeric (blue) and diluted-aggregated (green) PA as assessed by the Alamar blue assay (Fig. 5 and 6). Notably, 0.00125 wt% PA solutions resulted in viable cell numbers that were not significantly different to those cultures maintained in SFM alone for both monomeric and diluted-aggregated PA forms (Fig. 5 and 6 respectively). Higher concentrations of PA were less well tolerated; solutions of 0.01 wt% and 0.005 wt% both caused a loss of over 30% of cells compared to control (no PA) after only 72 hours in culture (ESI Fig. 1†).

Over seven days in culture, human corneal fibroblasts produced significantly greater total amounts of collagen both in monomeric and diluted-aggregated solutions of PA, at both 0.00125 wt% and 0.0025 wt% concentrations, where those cells in solutions of monomeric PA produced 6.7 μg (±0.2 μg) 7.4 μg (±0.8 μg) respectively, compared to 3.6 μg (±0.1 μg) in SFM alone (Fig. 5). Similarly, fibroblasts cultured in diluted-aggregated PA produced 7.8 μg (±0.2 μg) and 7.7 μg (±0.2 μg) of collagen respectively, compared to 4.4 μg (±0.2 μg) in SFM alone (Fig. 6). As the number of cells in each culture would undoubtedly influence the overall amounts of collagen synthesised, the total mass of collagen was normalized to viable cell number as determined by the alamar blue assay, revealing an approximately two-fold increase in collagen production per cell between fibroblasts in 0.00125 wt% PA and those cultured in SFM alone, in both monomeric PA (Fig. 5) and diluted-aggregated PA (Fig. 6) solutions, with a further, albeit less pronounced, increase in collagen per cell in 0.0025 wt% solutions compared to those at 0.00125 wt%.

**Fig. 7:**
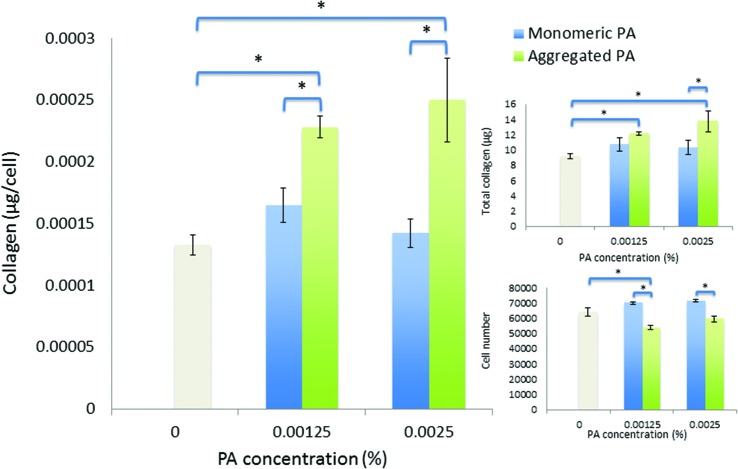
Viability and collagen synthesis by human corneal fibroblasts after culture for twenty-one days in monomeric PA and diluted-aggregated PA supplemented SFM.

Over an extended period of culture (21 days), human corneal fibroblasts cultured in PA solutions maintained good viability, where the least tolerated condition (0.00125 wt% diluted-aggregated PA) nevertheless retained >70% of the viable cell number observed in SFM controls. Fibroblasts in monomeric PA solutions showed improved viability compared to that observed over seven days, relative to controls. Cell numbers were not significantly different in the presence of 0.0025 wt% PA compared to 0.00125 wt% PA in either monomeric or diluted-aggregated PA solutions (Fig. 7). When directly compared over 21 days, values for both total mass of collagen produced and collagen per cell were shown to be significantly greater in response to diluted-aggregated PA solutions than monomeric PA at the same wt% (Fig. 7).

**Fig. 8:**
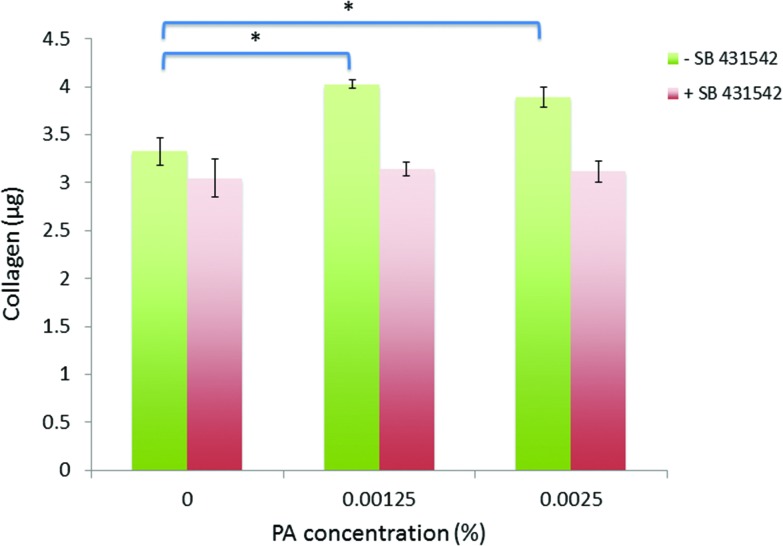
ALK receptor inhibition reduced the bioactivity of C_16_-YEALRVANEVTLN. Human corneal fibroblasts failed to respond to PA treatment in the presence of an ALK inhibitor (+SB 431542) compared to a significant response to PA treatment when exposed to the DMSO diluent alone (–SB 431542).

Fibroblasts treated with diluted-aggregated PA at these efficacious concentrations in the presence of the ALK receptor inhibitor (+SB 431542) were unresponsive to the PA, failing to synthesize more collagen over 7 days than those cells cultured without PA (no significant differences were observed between PA-treated and zero PA-treated control cultures), whereas those cells treated with the PA in the presence of the DMSO diluent only (–SB 431542) maintained their ability to produce significantly more collagen than the zero PA-treated control cultures (Fig. 8).

## Discussion

A peptide amphiphile (PA) has been designed based upon a biologically active peptide sequence derived from the C-terminal domain of lumican. This PA has been shown to self-assemble at 0.03 wt% to form bi-layered tape-like structures,^19^ however here we demonstrate that the beta sheet structures are maintained upon subsequent dilution to efficacious and biocompatible concentrations and importantly are stable over time in aqueous solution, suggesting that PA-aggregates formed at high concentration are kinetically trapped, resulting in no re-establishment of the monomeric state upon dilution. We believe such an observation has not previously been reported. This gives further credence to the application of PAs as a potential new class of bioactive molecule.

Low concentrations of PA in either monomeric or diluted-aggregated form are shown to be well tolerated by corneal fibroblasts in culture and C_16_-YEALRVANEVTLN strongly stimulates the synthesis of collagen. Of particular note, this enhancement is significantly greater in the case of the diluted-aggregated PA, suggesting a role of the secondary structure of this molecule in its biological activity. This was particularly apparent when the two conditions were directly compared over 21 days as shown in Fig. 7. It is worth noting that no similar direct comparison of the effects of monomeric and diluted-aggregated PA can be made from the differences shown between Fig. 5 and 6, as these experiments were run independently of each other using potentially variable primary cells derived from different sets of (*n* = 3) donor corneas. This variability may account for the observable difference in the negative controls in each of Fig. 5 and 6, which are otherwise identical conditions. The effect of C_16_-YEALRVANEVTLN on corneal fibroblasts was abrogated in response to the inhibition of ALK receptors, a known lumican binding site.^17^

As previously stated, the maintenance of the collagenous matrix of the corneal stroma by fibroblasts is essential to corneal transparency, and by extension to the function of the entire eye. Similarly the deposition of collagen by fibroblasts *in vitro* is of interest as a potential method for the production of native like tissue for the engineering of ‘organotypic’ cornea to eventually reduce or remove the demand for donor tissue. We have previously demonstrated the ability of cultured fibroblasts to synthesize a collagenous membrane with distinct similarities to stromal lamellae, and that can be recovered from culture, handled with relative ease, showing excellent potential for further investigation as a scaffold for the tissue engineering of an artificial corneal stroma.^3^ Tissue engineering processes such as this or the direct repair of diseased corneas *via* topical application, would likely benefit from an enhancement of extracellular matrix secretion by resident stromal fibroblasts. The peptide amphiphile C_16_-YEALRVANEVTLN showed good cyto-compatibility, as concentrations sufficient to induce a marked enrichment in collagen synthesis demonstrated little or no reduction in cell number, whilst higher concentrations capable of further enhancing collagen production still maintained cell numbers greater than 70% of those in standard medium. This compares favorably with Matrixyl™, a PA approved for commercial use in cosmetics for skin care. In similar conditions, Matrixyl™ was shown to reduce the number of viable fibroblasts by 70% or more.^7^ In terms of collagen production C_16_-YEALRVANEVTLN displays effects similar to those of Matrixyl™ on both corneal fibroblasts as shown here, and on dermal fibroblasts^19^ *in vitro*, but with seemingly less pronounced effects upon cell viability. Thus, as it is presumed that the effect of Matrixyl™ upon facial wrinkles^7,23^ is due to its collagen stimulating activity, C_16_-YEALRVANEVTLN may be equally or better suited to the commercial niche in skincare applications currently occupied by C_16_-KTTKS.

In addition to the observed effects of C_16_-YEALRVANEVTLN in solution, PAs have previously been used as coatings for culture surfaces or as scaffolds to mediate or instruct cell behavior.^3,5^ The PA investigated here may make a suitable addition to such systems, either individually to stimulate the production of collagen by cells for tissue engineering purposes, or as part of a multi-molecular system wherein a range of bioactive peptide motifs are presented in controlled ratios within the same supra-molecular structure. We have previously used a bi-molecular system incorporating RGDS presenting peptides along with a PA containing an inactive ‘spacer’ peptide head (ETTES)^3,5^ to reduce the amount of RGDS groups per unit area. The native ECM is formed of numerous fibrillar proteins, proteoglycans/glycosaminoglycans, and other molecules, each displaying a variety of motifs that are recognizable by cell surface receptors. The orientation and relative abundance of these motifs contribute to cellular recognition of the microenvironment and instruct cell behavior accordingly. It seems feasible, then, that PA superstructures that can present a range of precisely spaced and concentrated peptide motifs might be developed to mimic the instructional and regulatory properties of native tissue and thus perhaps reproduce *in vivo* niche conditions in *in vitro* culture systems and upon the surface of synthetic biomaterials. In this respect there is certainly a strong rationale to investigate the self-assembly and bioactivity and their inter-relationship of PAs containing bio-derived peptide sequences, as has been performed in this study.

These data support the potential of peptide amphiphiles for use as a novel class of bioactive molecules, in this instance for the support of tissue regeneration through stimulating the synthesis of the collagenous extracellular matrix. The *in vivo* repair of corneal injury extending beyond the epithelia into the stromal tissue requires the synthesis of new matrix material to replaced damaged tissue. This is dependent upon the activation of the quiescent fibroblast population. Within 6 hours of injurious insult, fibroblasts enter into the cell cycle, increase their organelle content and migrate towards the site of injury adopting a more fibroblastic phenotype.^24^ These cells then synthesise new extracellular matrix proteins and, over time, model the newly secreted matrix to reconstitute functional corneal tissue.^25^ Although responsive to stimulatory factors secreted by platelets, the avascular nature of corneal tissue means that many of the cues for this transition are provided by the cells of the corneal epithelium, and its basement membrane.^26^ This outside-in (*i.e.* from the external epithelial side of the tissue rather than internal vasculature) instruction of the fibroblast response to injury supports a potential role for topical application of factors that mediate the behaviour of fibroblasts. The ability to stimulate the production of extracellular matrix proteins, such as the collagens that form the bulk of this tissue, by the endogenous cell population may have potential in the treatment of diseases such as persistent corneal stromal ulcerations, and keratoconus, both sight threatening conditions characterised, in part, by ECM degradation.^27,28^

TGF-β is a common ligand against ALK receptors and its addition to cells *in vitro* has been shown to enhance the synthesis of collagen^29,30^ Similarly, lumican has been previously shown to be complementary to ALK receptors^17^ inducing receptor dimerization and activation of the SMAD dependent signaling pathway that can result in up-regulation of collagen synthesis.^31^ Thus it seemed probable that the peptide ‘head’ of the C_16_-YEALRVANEVTLN molecule would also interact with this pathway. Indeed, the effects of the PA upon collagen synthesis were considerably abrogated in the presence of the ALK receptor inhibitor (indicated by the loss of the statistically significant response observed in the absence of exogenous ALK inhibition) giving a nascent mechanistic insight into the biological action of this PA. Future studies could confirm these results using corneal fibroblasts from SMAD knockout mice.^32^

**Fig. 9:**
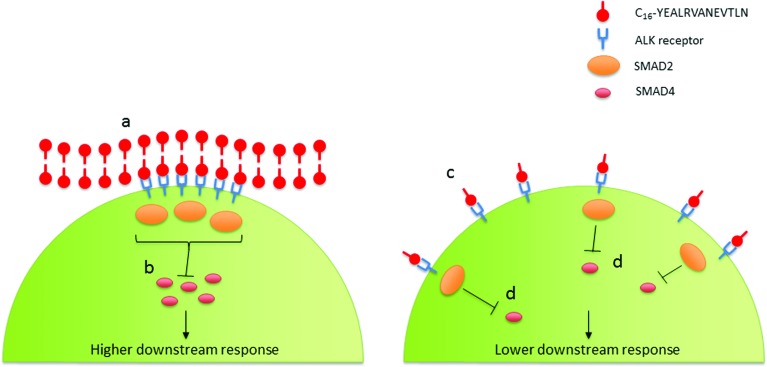
Possible localization of ALK receptors to PA nanotapes: Diluted-aggregated PA (a) presents a high regional concentration of a bioactive sequence (*e.g.* YEALRVANEVTLN) capable of binding to a transmembrane receptor (*e.g.* ALK) potentially resulting in a localized increase (b) in the activation of signal transducing proteins (SMAD2, SMAD4) and potentially greater downstream signalling, when compared to an equal concentration of monomeric PA solution, wherein the active sequence is presented to the cell surface in a more disperse fashion (c) leading to a less localized activation of signalling proteins (d) and thus comparatively lesser downstream signalling.

Interestingly the significance of the diluted-aggregated form of C_16_-YEALRVANEVTLN on collagen production may be dependent upon the enhanced activation of ALK receptors. The retained supra-molecular structure of the diluted-aggregated PA has the unique capacity to present a highly concentrated amount of the bio-active peptide to the cells. Dense peptides along the surface of the nanotape could lead to a localized concentration (many times higher than the overall concentration of PA in solution) at the cell surface. As such these regions of high ligand density could aid in the assembling and clustering of the ALK receptors resulting in a focal point for SMAD phosphorylation (Fig. 9). Achieving such a ‘critical mass’ of localized signaling may not be so readily achieved when using the same wt% PA solution in its monomeric form (as indeed our data suggests).

In summary, a peptide amphiphile has been designed based upon a biologically active peptide derived from the C-terminal domain of lumican. This PA self assembles at 0.1 wt% to form bilayered tape-like structures that are stable in aqueous solution and maintained upon subsequent dilution, is biocompatible at efficacious concentrations, and stimulates collagen synthesis by corneal fibroblasts. This stimulatory effect is enhanced by aggregation of PA into supra-molecular structures and the biological effects of this nanotape form may be mediated by ALK receptor activity. As such these findings demonstrate the cyto-compatibility and bioactivity of C_16_-YEALRVANEVTLN, reveal the influence of supra-molecular assembly and, overall, support the potential use of a self-assembling lumican-derived PA as a novel biomaterial, intended to promote collagen deposition for wound repair and tissue engineering purposes.

## Abbreviations

ALK: Activin receptor-like kinase
ANOVA: Analysis of variance
CAC: Critical aggregation concentration
CD: Circular dichroism
DMEM: Dulbecco's modified eagle's medium
DMSO: Dimethyl sulfoxide
ECM: Extracellular matrix
FBS: Fetal bovine serum
HSD: Honest significant difference
ITS: Insulin, Transferrin and Selenium
PA: Peptide amphiphile
SEM: Standard error of the mean
SFM: Serum-free media
TGF-β: Transforming growth factor-beta
