# Supplementary material for: Supra-molecular assembly of a lumican-derived peptide amphiphile enhances its collagen-stimulating activity
Source: Biomater Sci. 2015 Dec 2;4(2):346–54. doi: 10.1039/c5bm00428d (PMC4743677; doi:10.1039/c5bm00428d)
Supplement: Supplementary file 1 [file BM-004-C5BM00428D-s001.pdf]

This journal is © The Royal Society of Chemistry 2015

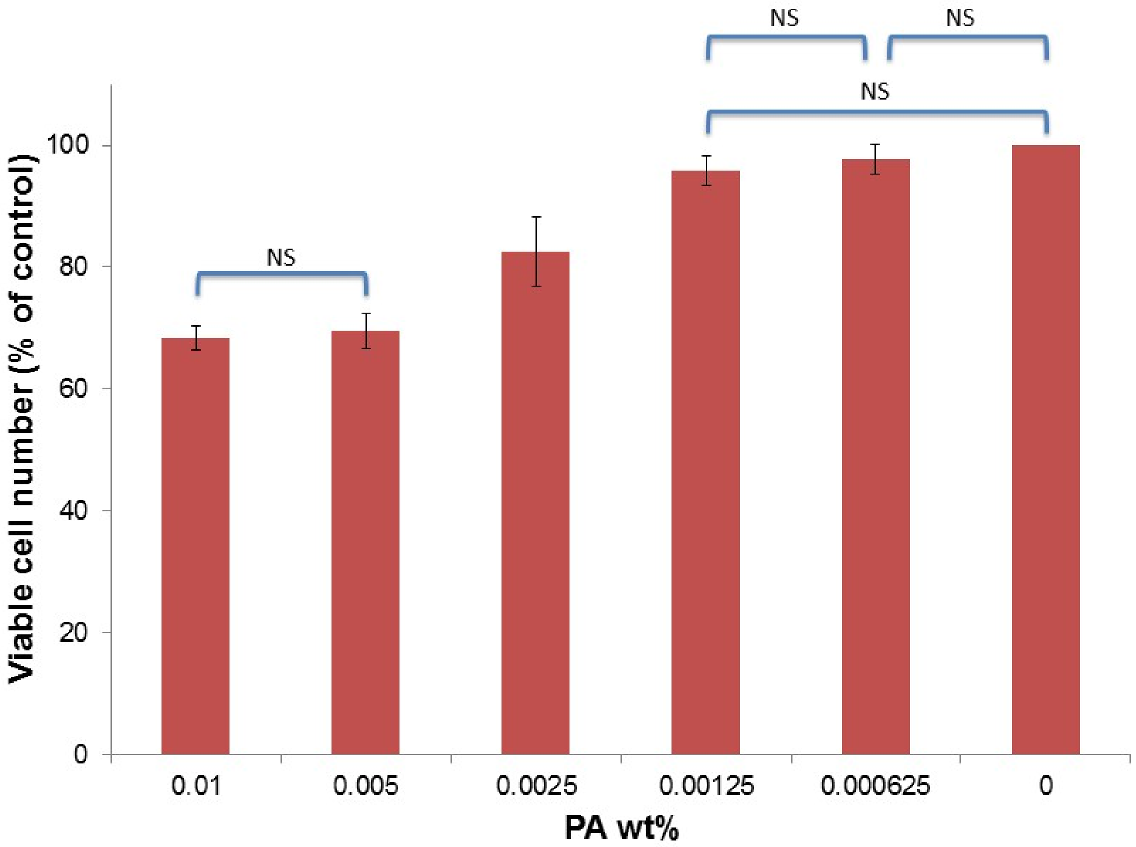

Supplementary Figure 1. Cytotoxicity of C_{16}-YEALRVANEVTLN. PA supplemented growth medium was well tolerated by human corneal fibroblasts up to concentrations of 0.0025 wt%, maintaining >80% of viable cell number compared to un-supplemented control medium over 72 hours in culture. Moreover, lower concentrations of PA (0.00125 wt% and 0.000625 wt%) showed no significant difference (NS) compared to un-supplemented medium (0 wt%) in terms of toxicity. No individual significant differences were also observed between 0.01 wt% and 0.05 wt%, 0.00125 wt% and 0.000625 wt%, as shown by Tukey's HSD test. All other individual comparisons were significantly different. An overall significant difference was revealed by ANOVA.
